# Iron incorporation both intra- and extra-cellularly improves the yield and saccharification of switchgrass (*Panicum virgatum* L.) biomass

**DOI:** 10.1186/s13068-021-01891-4

**Published:** 2021-03-04

**Authors:** Chien-Yuan Lin, Bryon S. Donohoe, Yannick J. Bomble, Haibing Yang, Manal Yunes, Nicholas S. Sarai, Todd Shollenberger, Stephen R. Decker, Xiaowen Chen, Maureen C. McCann, Melvin P. Tucker, Hui Wei, Michael E. Himmel

**Affiliations:** 1 Biosciences Center, National Renewable Energy Laboratory, Golden, CO 80401 USA; 2 Department of Biological Sciences, Purdue University, West Lafayette, IN 47907 USA; 3 National Bioenergy Center, National Renewable Energy Laboratory, Golden, CO 80401 USA; 4 Present Address: Joint BioEnergy Institute, Lawrence Berkeley National Laboratory, Emeryville, CA 94608 USA; 5 Present Address: Environmental Genomics and Systems Biology Division, Lawrence Berkeley National Laboratory, Berkeley, CA 94720 USA; 6 Present Address: South China Botanical Garden, Chinese Academy of Sciences, Guangzhou, 510650 China; 7 Present Address: Department of Chemical and Biological Engineering, University of Colorado Boulder, Boulder, CO 80309 USA; 8 Present Address: Division of Chemistry and Chemical Engineering 210-41, California Institute of Technology, 1200 East California Boulevard, Pasadena, CA 91125 USA

**Keywords:** Ferritin, Iron co-catalyst, Transgenic switchgrass, High-throughput hot-water pretreatment, Saccharification, Sugar release, Perls’ Prussian blue staining

## Abstract

**Background:**

Pretreatments are commonly used to facilitate the deconstruction of lignocellulosic biomass to its component sugars and aromatics. Previously, we showed that iron ions can be used as co-catalysts to reduce the severity of dilute acid pretreatment of biomass. Transgenic iron-accumulating Arabidopsis and rice plants exhibited higher iron content in grains, increased biomass yield, and importantly, enhanced sugar release from the biomass.

**Results:**

In this study, we used intracellular ferritin (FerIN) alone and in combination with an improved version of cell wall-bound carbohydrate-binding module fused iron-binding peptide (IBPex) specifically targeting switchgrass, a bioenergy crop species. The FerIN switchgrass improved by 15% in height and 65% in yield, whereas the FerIN/IBPex transgenics showed enhancement up to 30% in height and 115% in yield. The FerIN and FerIN/IBPex switchgrass had 27% and 51% higher in planta iron accumulation than the empty vector (EV) control, respectively, under normal growth conditions. Improved pretreatability was observed in FerIN switchgrass (~ 14% more glucose release than the EV), and the FerIN/IBPex plants showed further enhancement in glucose release up to 24%.

**Conclusions:**

We conclude that this iron-accumulating strategy can be transferred from model plants and applied to bioenergy crops, such as switchgrass. The intra- and extra-cellular iron incorporation approach improves biomass pretreatability and digestibility, providing upgraded feedstocks for the production of biofuels and bioproducts.

## Background

As the demand for sustainable energy and bioproducts increases along with the growth of the world population [1, 2], the supplies of improved bioenergy crops and alternative feedstocks to meet escalating demands are becoming important challenges [3–5]. Biomass feedstocks are also considered a readily available source to replace petroleum-based resources and provide energy, transportation fuels, and bioproducts, resulting in environmentally friendly alternatives for reducing net long-term carbon dioxide emissions [6–8]. In contrast to first-generation biofuels that can affect food supplies, the second generation of biofuels relies primarily on lignocellulosic biomass [9]. With the advancement of interdisciplinary expertise in “green” technology for second-generation biofuels, concepts for the lignocellulosic biorefinery have recently emerged and are defined as the sustainable processing of biomass and conversion to a wide range of bioenergy products (i.e., energy, heat, and biofuels) and other bioproducts (i.e., supplement, chemicals, and/or materials) [10–12].

Several non-food plant species have been designated as dedicated lignocellulosic biomass crops based on their high yields and/or rapid growth [9, 13–15]. Examples are switchgrass [16, 17], *Miscanthus* [18], sorghum [19], *Populus* [20], and willow [21]. The main structural component of lignocellulosic biomass is the secondary cell wall (SCW), which consists of 40 to 50% cellulose, 15 to 25% hemicellulose, and 20 to 25% lignin [22, 23]. To utilize lignocellulosic biomass as a starting material, the biomass often requires pretreatment to loosen the interweaving networks of lignocellulosic fibers via thermal, chemical, or electrochemical processes [24], such as dilute sulfuric acid [25], alkaline [26], ammonia fiber explosion (AFEX) [27], steam explosion [28], liquid hot water [29] or pulsed electric field (PEF) [30, 31]. After pretreatment, the natural lignocellulosic networks are disrupted, which results in partial cell wall deconstruction, including wall delamination and defibrillation. These pretreated and modified walls are more readily attacked by lytic saccharification enzymes [32, 33]. However, biomass recalcitrance still poses a challenge for the cost-effective breakdown of plant cell walls. The current barriers for the use of all known pretreatment technologies include the high energy inputs/waste outputs and complex nature of the resulting pretreated biomass and liquors [34–36].

Switchgrass is a warm-season perennial C4-type grass species, which is native to central and north American [37]. Switchgrass has several advantages compared to other bioenergy crops, such as lower fertilizer requirement, higher yield potential, good pest/disease tolerance, better water/nutrient use efficiency, good growth on marginal lands, and resilience to biotic and abiotic stresses [38–43]. Switchgrass has an extensive root system, which can provide excellent soil conservation and carbon sequestration while being compatible with conventional farming practices [44]. It has been proposed as a potential dual-purpose crop for both bioenergy and forage [45]. However, switchgrass biomass has higher recalcitrance and requires higher severity pretreatments than other lignocellulosic feedstocks such as corn stover, which is due to the unique structure of switchgrass cell walls [46–48].

With the advances in genomic technologies, it is now possible to discover natural variations with adaptive traits that are beneficial for bioenergy or bioproduct production by next-generation sequencing [49]. However, to rapidly and directly improve the quality of plant biomass, plant genetic engineering remains the most effective and efficient approach [50], especially considering the high degree of self-incompatibility of switchgrass, which makes it challenging to retain quantitative traits under conventional breeding and selection methods [51]. Many attempts have been conducted in planta to reduce the cell wall recalcitrance of plant biomass. Target genes include those involved in the lignin biosynthetic pathway [52–54], lignin polymerization [55, 56], lignin manipulation [57–59], and polysaccharide content [60–63]. Over the last decade, genetic modification of switchgrass has successfully improved the quality of switchgrass by reducing lignin content, modifying lignin structure, enhancing fermentable sugar release for better saccharification efficiency; as well as by increasing the biomass yield [64–76].

Biomass recalcitrance is the primary barrier to the efficient and economical production of advanced biofuels or value-added bioproducts [77]. Instead of targeting the biosynthesis of a specific plant cell wall component that might affect plant growth, we developed and patented a promising approach to increase cell wall pretreatability using iron ions as co-catalysts [78]. We identified several essential factors that contributed to iron ion-enhanced efficiency during dilute acid pretreatment of biomass and elucidated the enhancement mechanisms [79]. Further, we demonstrated the successful accumulation of iron in Arabidopsis plants by overexpressing soybean ferritin intracellularly (referred to as FerIN) [80], while post-harvest stems of Arabidopsis plants showed enhanced pretreatability (i.e., released 13–19% more glucose/xylose than EV control). Similarly, Yang et al. developed a novel strategy for in planta accumulation of iron in Arabidopsis and rice using a cell wall targeted iron-binding peptide (IBP) [81]. Our results showed enhanced iron accumulation and improved biomass conversion with 20% more glucose and 15% more xylose release than controls [81]. Delivery of ferritin extracellularly into the plant cell wall (referred to as FerEX) resulted in increased biomass yield and even higher pretreatability and digestibility (released 21% and 34% more glucose and xylose, respectively) than the FerIN Arabidopsis plants [82]. Moreover, this in planta iron accumulation is valuable when considering its use for iron biofortification for human nutrition. This point was demonstrated in rice grains by cell wall targeted-IBP overexpression [81] and in wheat by increasing metal chelator biosynthesis [83].

Here, we translate these discoveries from model plants to the bioenergy crop, switchgrass. First, we introduced ferritin protein intracellularly under constitutive control of the 35S-CaMV promoter in switchgrass (FerIN). Second, we stacked an improved version of the extracellular iron-binding peptide (IBPex) into the FerIN-expressing switchgrass [81]. The resulting transgenic switchgrass lines were characterized with regard to transgene expression, biomass yield, and digestibility and pretreatability of stems. We cite the following evidence: (1) Using protein engineering, we generated an improved version of native IBPex with improved iron-binding ability by combining four tandem repeats of the IBP peptide (now 4xIBP). (2) The presence and expression of transgenes (ferritin and IBP) were confirmed by genomic DNA PCR, Southern blot, qRT-PCR, and western blot analyses. (3) The phenotype of the transgenic switchgrass was significantly increased in both height (up to ~ 15% in FerIN transgenic and ~ 30% in FerIN/IBPex) and yield (~ 65% in FerIN transgenic and ~ 115% in FerIN/IBPex). (4) Improved in vitro iron-binding activity and in planta iron-accumulating ability were observed in both FerIN and FerIN/IBPex transgenic switchgrass. (5) Compared to the EV control, the FerIN transgenic switchgrass showed enhanced pretreatability by releasing ~ 14% more glucose, whereas FerIN/IBPex switchgrass releases up to 24% more glucose. In conclusion, this iron incorporation strategy using iron-binding protein/peptide with spatiotemporal optimization can positively impact the quality of switchgrass biomass.

## Results

### Design of an improved SP-CBM-IBP with enhanced iron-binding ability

To overcome the recalcitrance of switchgrass, we aimed to design an improved version of switchgrass based on the success of our previous study using model plants [81]. We generated a unique signal peptide-carbohydrate-binding module-iron-binding peptide (SP-CBM-IBP), with enhanced iron-binding ability, for genetic engineering of switchgrass. We swapped the SP from dicot extensin protein with the rice glycine-rich protein (GRP) [84]. The extracellular secretory nature of this monocot SP has been successfully demonstrated in monocot plants, such as switchgrass [85] and sorghum [86]. Then, we retained CBM11 for cell wall targeting because it is an effective and preferential delivery system [81]. Third, we also utilized the IBP fragment for iron-binding, which binds iron across a wide range of pH and is known to facilitate iron accumulation in plants [81]. Finally, to boost the iron-binding ability of SP-CBM-IBP, we generated tandem repeats of the IBP fragments to increase the iron-binding capacity of the polypeptide. Two DNA fragments were synthesized encoding the triple fusion polypeptides, which were SP_GRP_-CBM11-IBP and SP_GRP_-CBM11-4xIBP (Additional file 1: Figure S1).

**Fig. 1:**
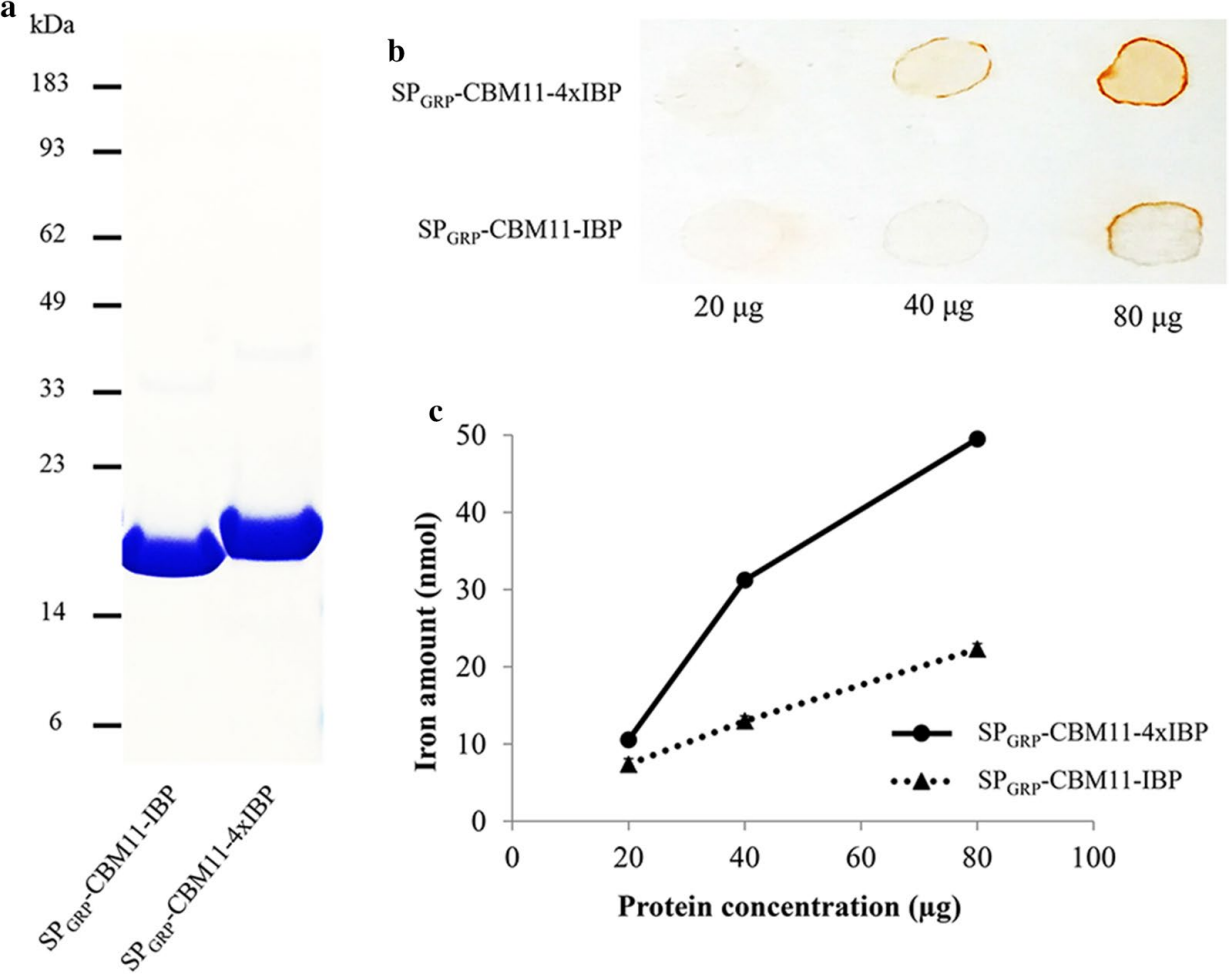
Protein purification of iron-binding fusion proteins and iron-binding assay. **a** The SDS-PAGE analysis of SP_GRP_-CBM11-IBP and SP_GRP_- CBM11-4xIBP proteins purified from recombinant *E. coli* cells. **b** Comparison of the iron-binding ability of iron-binding fusion proteins at pH 5.5 on a PVDF membrane. **c** Comparison of the iron-binding ability of iron-binding fusion proteins at pH 7.0 using light absorbance at 510 nm

To evaluate the iron-binding ability of the optimized polypeptides, we cloned the synthesized DNA fragments of SP_GRP_-CBM11-IBP and SP_GRP_-CBM11-4xIBP into the *E. coli* expression vector, and expressed them individually in *E. coli*. The expressed fusion proteins were purified and resolved by SDS-PAGE, which showed that SP_GRP_-CBM11-IBP is smaller than SP_GRP_-CBM11-4xIBP. This result matches the predicted molecular weights of 19.4 kDa and 22.5 kDa, respectively (Fig. 1a).

The purified fusion proteins were used for an in vitro iron-binding assay using two different pH ranges to compare their iron-binding ability using colorimetric iron-binding methods (Additional file 1: Figure S2). At pH 5.5, similar to cell wall pH, SP_GRP_-CBM11-4xIBP stained more intensely on the membrane than SP_GRP_-CBM11-IBP, when 40 or 80 μg of recombinant protein was used (Fig. 1b), indicating the improved iron-binding ability of SP_GRP_-CBM11-4xIBP. At pH 7, similar to cytosolic pH, the SP_GRP_-CBM11-4xIBP showed higher iron binding ability, which is about ~ twofold when 40 μg of recombinant protein was used and ~ 2.5-fold with 80 μg of SP_GRP_-CBM11-4xIBP (Fig. 1c). These results demonstrated successful enhancement of the iron-binding capacity by increasing the tandem repeats of IBP fragment using SP_GRP_-CBM11-4xIBP polypeptide (hereafter called IBPex).

### Production of transgenic switchgrass and molecular analyses

Two DNA constructs were prepared: (1) intracellular ferritin (FerIN), and (2) FerIN stacking with cell wall targeting IBPex (FerIN/IBPex). The DNA constructs were transformed into *Agrobacterium tumefaciens* EHA105 individually. The presences of transgenes (hygromycin phosphotransferase *(hph)*, *IBP*, and ferritin, Additional file 1: Figure S3) were confirmed by PCR in the *Agrobacterium* transformants using corresponding primer sets (Additional file 1: Table S1A).

**Fig. 2:**
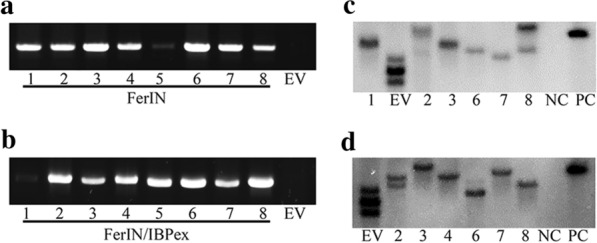
Molecular analyses of the engineered switchgrass transgenics using genomic DNA PCR and Southern blot (SB). **a** Genomic DNA PCR of FerIN transformed transgenic switchgrass. **b** Genomic DNA PCR of FerIN/IBPex transformed transgenic switchgrass. **c** SB of the FerIN transformed transgenic switchgrass. **d** SB of the FerIN/IBPex transformed transgenic switchgrass. For SB, the probe for detecting the *hph* gene was used. *EV* empty vector, *NC* negative control, *PC* positive control. EV was obtained from Lin et al. [43]

Using our previously established switchgrass transformation protocol [43], eight independent transgenic lines of each construct were successfully obtained within 6 months. The primary screening of the putative transgenics was conducted using genomic DNA PCR to detect the presence of the transgenes. Seven of the eight transgenic lines showed positive signals for the FerIN construct (Fig. 2a), as well as for FerIN/IBPex (Fig. 2b). Reverse transcription PCR (RT-PCR) was then used to verify the presence of the transgenes and eliminate the false-positive result from genomic PCR. The RT-PCR results of the FerIN transgenics showed only the transcripts of the ferritin, but not IBP (Additional file 1: Figure S4, left). Transcripts of both ferritin and IBP can be detected in the FerIN/IBPex transgenic plants (Additional file 1: Figure S4, right), which is consistent with the genomic DNA PCR (Fig. 2a, b). The integration of T-DNA was also examined by Southern blots using our previously optimized procedure [43], and positive hybridization signals were detected in all selected transgenic plants (Fig. 2c, d).

### Expression of FerIN and IBPex in transgenic switchgrass lines

**Fig. 3:**
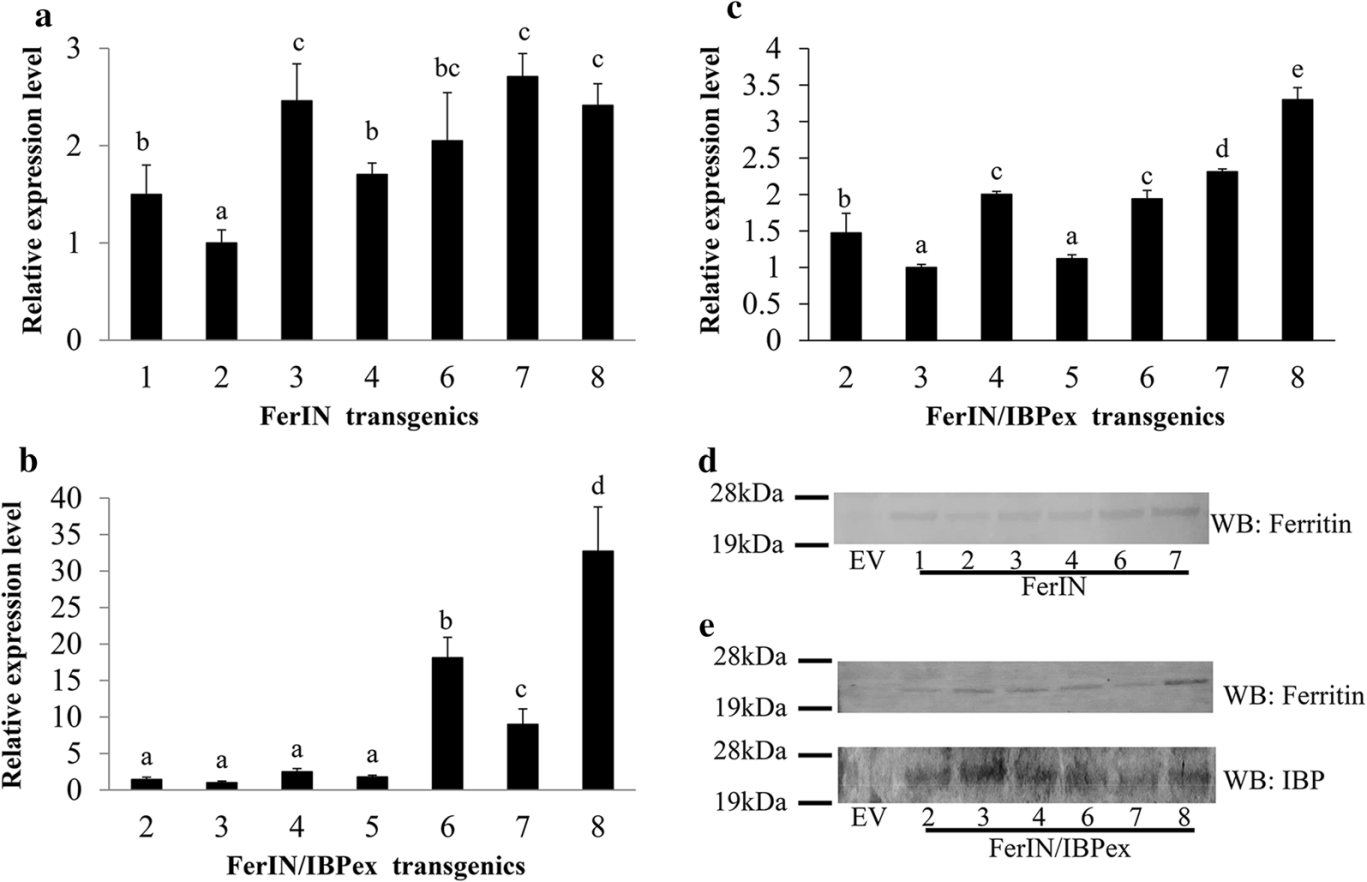
Determination of transgene expression by transcriptional (qRT-PCR) and western blot (WB) analysis. **a** The ferritin transcript level of FerIN transgenic switchgrass. **b** The ferritin transcript level of FerIN/IBPex transgenics. **c** The IBP transcript level of FerIN/IBPex transgenics. **d** WB analysis of ferritin expression in FerIN transgenics. **e** WB analysis of IBP and ferritin expression in FerIN/IBPex transgenics. For qRT-PCR, the transgenics with the lowest expression level of ferritin or IBP was set to 1. Data are presented as the mean (± standard error, SE) of three replicates and bars represented by different letters are significantly different at *p* < 0.05 between lines analyzed by one-way ANOVA with Tukey’s test. For WB, the corresponding antibodies used are indicated on the right side of the WB result

The expression levels of ferritin and IBP in transgenic switchgrass lines were tested first by real-time quantitative RT-PCR (qRT-PCR). Compared to the lowest expression level of the transgenes among the transgenic lines tested, the expression level of ferritin varied up to three-fold in FerIN lines and up to 35-fold in FerIN/IBPex lines (Fig. 3a, b); whereas the level of IBP is ~ 3.5-fold in FerIN/IBPex lines (Fig. 3c). Using total soluble proteins extracted from the stem tissues of transgenic switchgrass lines, we were able to detect ferritin (~ 26 kDa) in the FerIN transgenic lines (Fig. 3d); as well as in the FerIN/IBPex transgenic lines (Fig. 3e, top), by western blot analysis, using chicken IgY polyclonal antibody against the synthesized soybean ferritin peptide. The expression of the IBP was also detected as an expected ~ 22.5 kDa band using the commercial monoclonal His-tag antibody against the C-terminal 6xhis-tag epitope of IBPex (Fig. 3e, bottom).

Based on the high transcriptional levels and successful protein expression of the transgenes, three independent lines for each of the two constructs (FerIN and FerIN/IBPex) were selected for further characterization: lines 3, 6, and 7 of FerIN and lines 6, 7, and 8 of FerIN/IBPex transgenic plants (Fig. 3).

### Plant height and biomass yield of the FerIN and FerIN/IBPex transgenic switchgrass

**Fig. 4:**
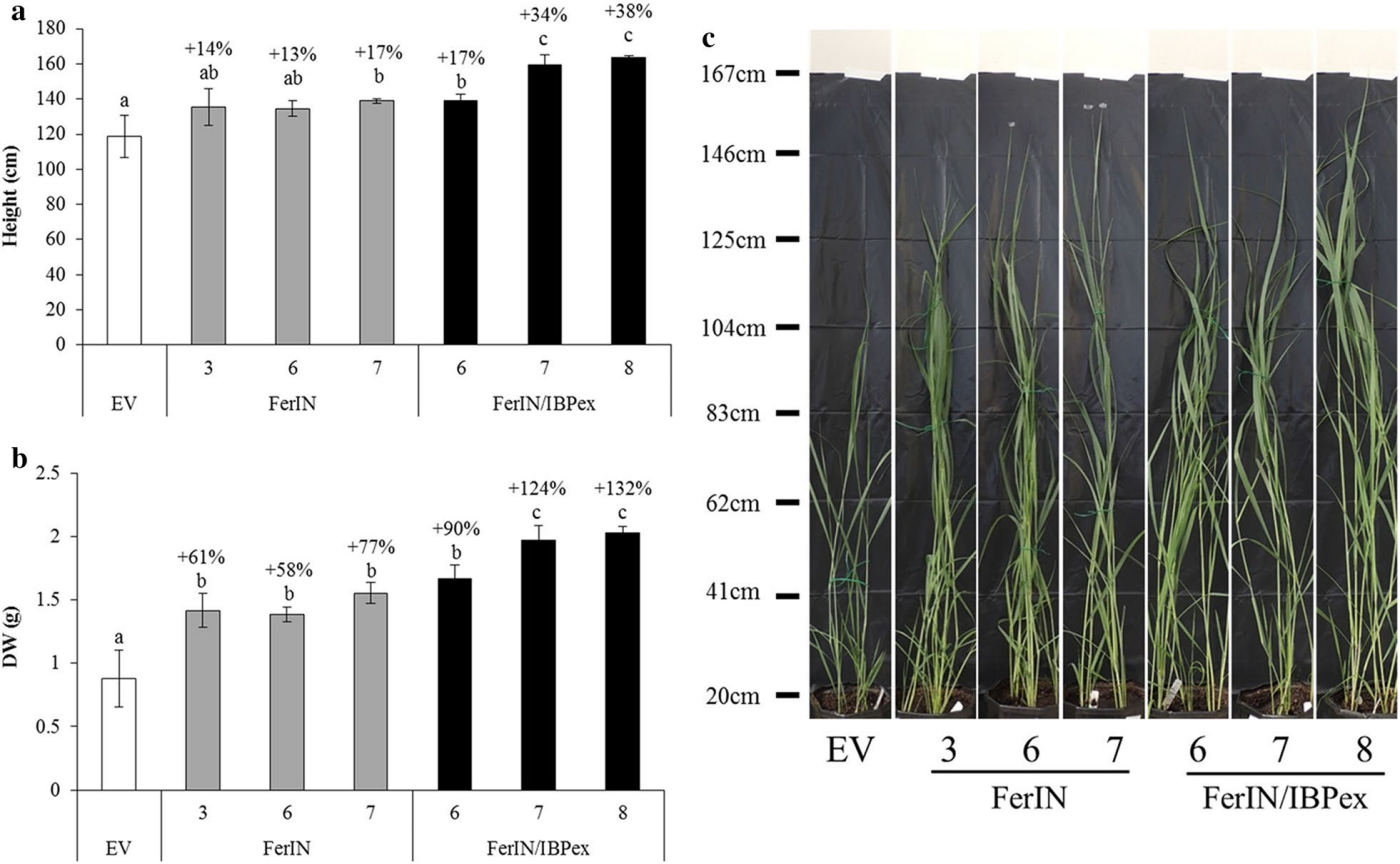
Growth, biomass yield, and phenotype comparison between EV, FerIN, and FerIN/IBPex transgenic switchgrass. **a** The height of the transgenic plants. **b** The biomass yield of the transgenic plants. **c** The representative growth phenotype of the transgenic yield of the transgenic plants. Data are presented as the means (± SE) of three replicates. The percentage values on the top of bars represent the % increase in the transgenic lines compared to the empty vector (EV) control plants. Different letters are significantly different at *p* < 0.05 between lines analyzed by one-way ANOVA with Tukey’s test. *DW* dry weight

Compared to the EV control, the average height of 4-month-old greenhouse-grown plants was 15.0 ± 1.1% higher in FerIN transgenic lines and 29.8 ± 6.4% higher in FerIN/IBPex lines (Fig. 4a). Especially notable is the FerIN/IBPex-8, which showed a 38% increase in height (Fig. 4a).

We also observed a remarkable improvement in the average biomass yield of the transgenic lines. FerIN transgenic switchgrass showed 65.8 ± 5.9% higher yield than the EV control, whereas the FerIN/IBPex plants increased even further in yield to 115.8 ± 12.8% (Fig. 4b). All FerIN transgenics (FerIN-3, -6, and -7) were significantly improved in weight (i.e., 58% to 77%). The FerIN/IBPex transgenic plants showed more improvement than did the EV control and FerIN transgenics, which was up to ~ 132% increase in yield compared to the EV control (FerIN/IBPex-8 in Fig. 4b). The yield improvement may result from the increased number of tillers per transgenic plants in FerIN transgenic plant and a combination of improved plant height, increased stem diameter, and the number of tillers per transgenic plants in FerIN/IBPex (Fig. 4c).

### Shoot iron content of transgenic plants

**Fig. 5:**
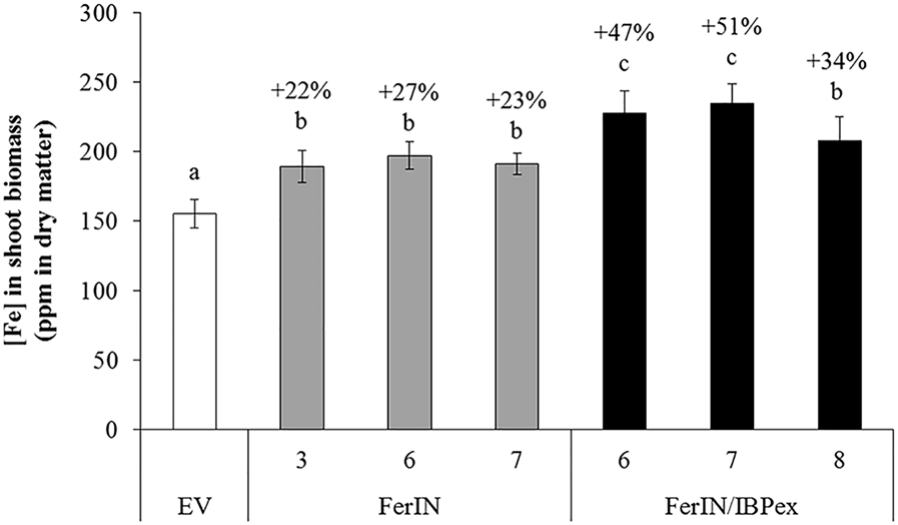
Iron contents in shoot biomass from switchgrass transgenics. Data are presented as the mean (± SE) of five replicates. The percentage values on the top of bars represent the % increase of Fe contents in the transgenic lines compared to the EV control. Different letters are significantly different at *p* < 0.05 between lines analyzed by one-way ANOVA with Tukey’s test

In planta iron accumulation was measured in the shoot biomass of three representative transgenic lines per construct transformation. ICP-OES analysis of nitric acid-digested biomass showed that iron contents in the shoot tissues of FerIN transgenic plants (189 to 197 ppm in dry matter) were 22% to 27% higher than that of the transgenic EV control plants (155 ppm in dry matter) under normal growth conditions with distilled H_2_O-watering (Fig. 5). Iron content in the shoots of the FerIN/IBPex transgenic plants (207 to 235 ppm in dry matter) was also approximately 34% to 51% higher than that of the EV control plants (Fig. 5), indicating that the stacking of FerIN gene with IBPex gene did lead to higher in planta iron-accumulating ability compared to the FerIN gene alone in switchgrass.

### Iron-binding ability of transgenic plants at the protein extract and tissue levels

**Fig. 6:**
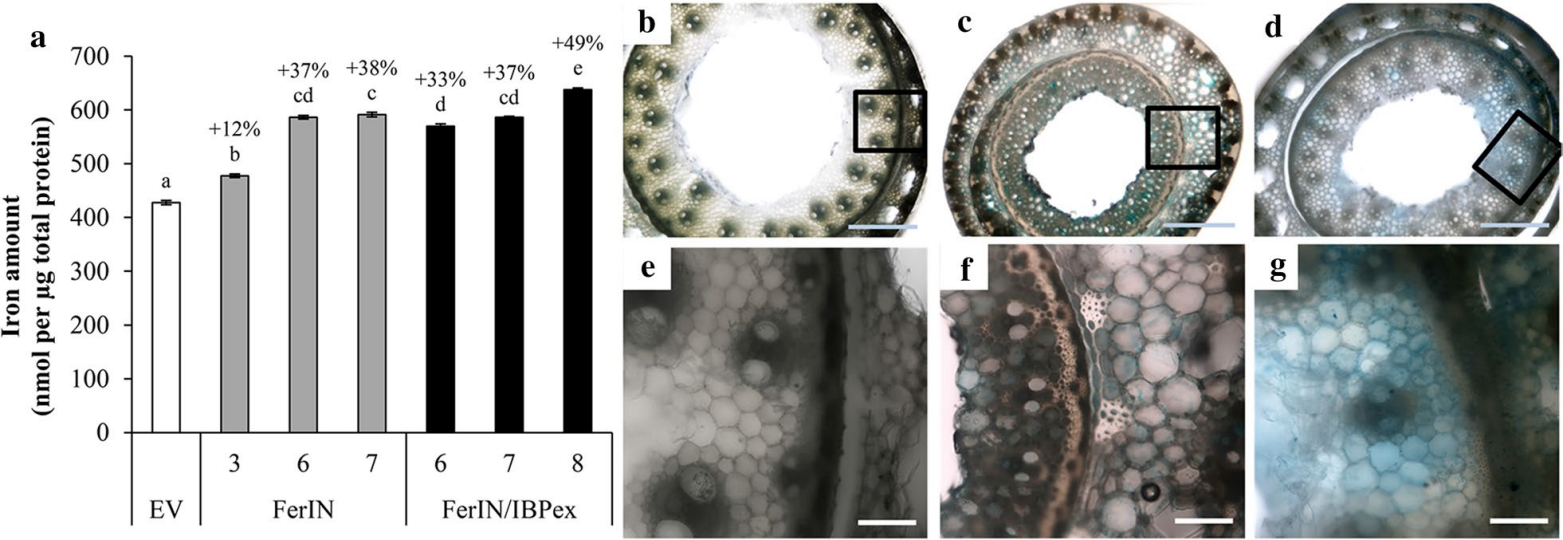
Iron-binding ability among representative transgenic plants at the protein extract and tissue levels. **a** Iron binding assay using crude ste, extract of EV, FerIN, and FerIN/IBPex. Data are presented as the mean (± SE) of three replicates. The percentage values on the top of bars of transgenic lines represent the % increase of iron-binding ability in the transgenic lines compared to the EV control. Different letters are significantly different at *p* < 0.05 between lines compared by one-way ANOVA with Tukey’s test. **b**–**g** Prussian blue staining of the switchgrass stem cross-sections. **b** EV. **c** FerIN transgenic lines. **d** FerIN/IBPes transgenic lines. **e** to **g** are higher magnification images of **a** to **c**. Scale bars in **b**–**d** are 300 µm; scale bars in **e**–**g** are 50 µm

We next investigated the iron-binding ability of the engineered switchgrass at the protein extract and tissue levels using biochemical and imaging analyses. Compared to the EV control, the iron-binding abilities of crude protein extracts from stems of FerIN and FerIN/IBPex transgenic lines were significantly enhanced (Fig. 6a). The iron-binding ability was improved by 12% in FerIN-3, and FerIN-6 and FerIN-7 lines showed 37% to 38% increases in iron binding ability, respectively (gray bars in Fig. 6a). For FerIN/IBPex transgenics, the iron-binding abilities were improved by 33% to 37% in FerIN/IBPex-6 and -7, and up to 49% increased in FerIN-8 (black bars in Fig. 6a).

Perls’ Prussian blue staining used to localize iron in cross-sections of stem tissues [80–82] by optical stereomicroscopy. In the EV control, the blue signals cannot be detected either within plant cell or on the cell wall (Fig. 6b, e); in contrast, we observed blue staining in the stem sections of FerIN and FerIN/IBPex (Fig. 6c, d). At higher magnification, in the stem of FerIN transgenics, blue staining was mostly in the cytosol (Fig. 6f), whereas in FerIN/IBPex lines, the blue stain was observed both in the cytosol and cell walls (Fig. 6g).

### Hot-water pretreatment and co-saccharification of the transgenic switchgrass biomass

We used the hot-water pretreatment and co-saccharification method to evaluate the effectiveness of our iron accumulation strategies, FerIN and FerIN/IBPex, in enhancing the cell wall deconstruction in switchgrass biomass. The hot-water pretreatment is a greener technology that not only benefits the environment but also avoids the corrosion effect of dilute acid to the reactor and eliminates the downstream step of neutralizing the pretreated biomass residue before saccharification.

**Fig. 7:**
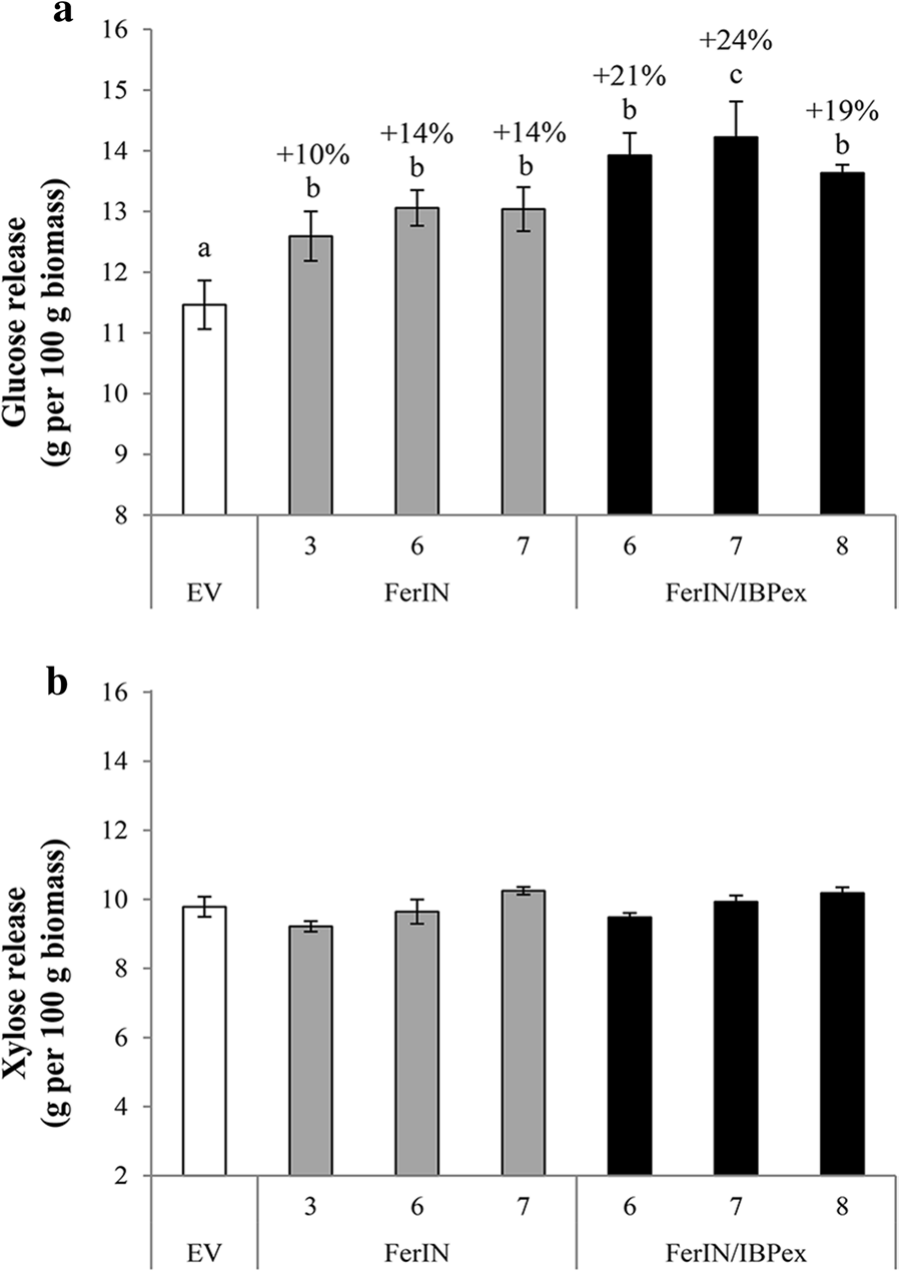
Pretreatability and digestibility of shoot biomass from switchgrass transgenics. **a** Total glucose release after the hot-water pretreatment and co-saccharification of shoot biomass. **b** Total xylose release after the hot-water pretreatment and co-saccharification. The percentage values on the top of bars of transgenic lines represent the % increase of released sugar in the transgenic lines compared to the EV control. Data are presented as the mean (± SE) of nine replicates. Different letters are significantly different at *p* < 0.05 between lines compared by one-way ANOVA with Tukey’s test

The results showed enhanced glucose release for both FerIN and FerIN/IBPex transgenic lines (Fig. 7). For the FerIN plants, the glucose release was increased approximately 10% to 14% compared to the EV control; whereas for the FerIN/IBPex plants, glucose release was enhanced further (i.e., 19% to 24%) (Fig. 7a). In contrast to glucose release, xylose release does not change in all the transgenic lines (Fig. 7b), which can be explained by the fact that, in general, the xylan in the plant cell wall is much more exposed, i.e., more easily degraded to sugar monomers (i.e., less recalcitrant) than cellulose [87]. Thus, the xylose release is very high to begin with, so there is not likely much room for further improvement; the effect of iron accumulation on the pretreatability and digestibility was more prominently reflected on the more recalcitrant part, cellulose.

## Discussion

### Improved iron binding ability of SP-CBM-IBP by protein engineering

Recently, a successful approach using a small signal peptide for extracellular secretion and cell wall targeting towards iron accumulation was achieved by Yang et al. [81], which showed improved biomass pretreatability and sugar yield in both dicot (Arabidopsis) and monocot (rice). This strategy for iron accumulation used a triple fusion polypeptide, which comprised an extensin signal peptide (SP_EXT_) from *Nicotiana plumbaginifolia* for extracellular secretion [88], the CBM11 from *Clostridium thermocellum* for cell wall targeting [89] and a synthetic blood IBP from porcine for iron-binding [90]. In this study, we investigated whether or not we can improve iron accumulation and improve the cell wall pretreatability and saccharification of switchgrass by the intracellular ferritin (FerIN) strategy and by stacking with cell wall targeting strategy using secretory iron-binding peptide.

First, we modified the SP-CMB-IBP approach used by Yang et al. [81] based on our previous experience from FerEX Arabidopsis [82]. We replaced the secretion signal peptide from SP_EXT_ with rice GRP (SP_GRP_), as it had been suggested that the secretory property of rice SP_GRP_ can improve the protein expression of β-glucuronidase (GUS) in monocot plants, which demonstrated the higher intensity of blue coloration in both sorghum and switchgrass [85, 86] and higher GUS enzyme activity in apoplastic fluids of transgenic sorghum [86]. The CBM11 moiety was retained for cell wall targeting, considering that the SP_EXT_-CBM11 was determined to have the strongest cell wall-localized fluorescence signal among the five identified CtCBMs [81]. In addition, we optimized the iron-binding ability of IBP by generating a tandem repeat construct of IBP (4xIBP). The resulting “upgraded” version of SP_GRP_-CBM11-4xIBP was successfully expressed, and up to 2.5-fold improvement in iron-binding ability relative to SP_GRP_-CBM11-IBP was observed (Fig. 1). Although we successfully increased the iron-binding ability of the “improved” IBP design, a non-linear relationship between the number of IBP repeats and the fold-change in iron-binding capacity implies a limitation in our stacking approach, which may be due to a physical or stereochemical barrier for iron-binding (Fig. 1c).

### Genetic iron incorporation improves switchgrass biomass yield

Using our recently optimized switchgrass transformation protocol by *Agrobacterium*-mediated transformation, we successfully obtained FerIN and FerIN/IBPex switchgrass transgenics with high selection efficiencies in six months (87.5%, Fig. 2a, b), which is similar to our previously established range of 50 to 100% [43]. In addition, from Southern blots, a low integration number (one to two copies) of the transgenes was observed in the genome of transgenic switchgrass (Fig. 2c, d), which is also similar to the range of *Agrobacterium*-mediated genetic transformation [91, 92]. In our experience producing transgenic switchgrass, the primary screening using genomic DNA PCR is sufficient for selecting positive transgenic plants. The contamination of bacterial DNA seems negligible if the execution of genomic DNA extraction follows the manufacturer’s protocol because the molecular analyses of transgene expression are consistent, including qRT-PCR, western blot, and RT-PCR analyses (Fig. 3 and Additional file 1: Figure S4).

Previously, the intracellular ferritin (FerIN) Arabidopsis showed improved performance (i.e., 13 to 19% greater sugar release than EV control plants) [80]. In contrast, the characteristics of improved plant growth, enhanced pretreatability, and enzyme digestibility with boosted sugar release (21% more glucose, and 34% more xylose) were observed when using the extracellular ferritin (FerEX) approach [82]. Moving away from model plants (Arabidopsis and rice) in this study, we successfully transferred the iron incorporation strategy to the bioenergy crop platform, switchgrass. Similar to the result of FerIN Arabidopsis, the increase in biomass yield of FerIN switchgrass is mostly from the increased number of tillers (Fig. 4c) [80]. When stacking with IBPex in FerIN switchgrass, the cell wall targeting iron accumulation improved plant growth and development significantly (Figs. 4 and 5), which is consistent with the previous discovery when directing iron accumulation extracellularly [82].

Ferritin is a highly conserved protein important for iron storage and plays an essential role for iron homeostasis in animals, plants, and microorganisms. Overexpressing soybean ferritin in tobacco under a 35S promoter can enhance ferric chelate reductase activity, iron transport in the root, and photosynthesis, resulting in increased plant height and fresh weight [93]. The presence of ferritin in transgenic plants can also protect plants from free iron toxicity and photoinhibition while reducing oxidative stress [94]. In addition, it is well-known that iron (Fe) is also an essential micronutrient and often a limiting factor for higher biomass production and quality [95], while Fe deficiency in plants often results in severe chlorosis [96]. Due to the physiological importance of iron, improved plant growth and development in ferritin-overexpressing transgenic plants have been generally observed in several studies [97–99]. We hypothesized the enrichment of iron supply in the plant body by our iron incorporation strategy may affect the metabolism of oxidative stress, the function of chlorophyll and, hence, result in the superior growth outcomes and improved overall growth of our transgenic switchgrass plants.

### Genetic iron incorporation improves glucose yield from switchgrass biomass after hot-water pretreatment (HWP)

Different from our previously generated iron-accumulating transgenic plants, the improvements in switchgrass biomass saccharification using iron ions as co-catalysts reveals specificity towards glucose but not xylose [80, 82]. The results of pretreatability and digestibility of the FerIN switchgrass are 10 to 14% increases in glucose enzymatically released relative to the EV control, while FerIN/IBPex plants have 19–24% more glucose enzymatically released than the EV (Fig. 7). The observed glucose yield came from unwashed biomass after enzyme hydrolysis. It has been shown that there is negligible readily soluble glucose released from untreated switchgrass biomass [100].

A possible explanation is the fact that in general the xylose part of the plant cell wall is easier to break down into sugar monomers (i.e., less recalcitrant) than the cellulose part under the pretreatment conditions, thus the effects of iron accumulation on the pretreatability and digestibility were more prominently reflected on the more recalcitrant part, i.e., cellulose part of cell wall [101, 102]. Such observation highlights the commonality as well as the difference among plant species in implementing the in planta iron-accumulating strategy. Further improvement of the iron-incorporated switchgrass biomass could be made by tackling the release of xylose via introduction of thermostable xylanase [103]. Another perennial wild grass species with promise for bioenergy applications, *Miscanthus*, has recently been sequenced [104], and it is worthwhile to investigate whether our iron incorporation strategy can have a similar or better effect to reduce the grass biomass recalcitrance of *Miscanthus*.

### Genetic incorporation of iron coupling with HWP can be an economical and environmentally friendly approach for downstream biorefinery applications

Hot-water pretreatment (HWP) is a popular thermal treatment for lignocellulose biomass and has several benefits for the biorefinery including the following: (1) no additional chemical inputs except water; (2) little erosion on equipment; (3) low electricity usage; (4) reduced production of inhibitors to enzyme hydrolysis or fermentative microorganisms [29]. Most importantly, HWP requires lesser chemical/energy/equipment cost, resulting in cheaper biological conversion of lignocellulosic biomass.

We have demonstrated that iron ion co-catalysts can reduce the barriers of biomass pretreatment and facilitate lignocellulosic biomass conversion by enzymatic hydrolysis [78, 79]. The main shortcoming of previous versions of our technique was the application of excess iron ions before pretreatment. The exogenous iron input can result in additional equipment costs, water usage, and waste disposal, while the effectiveness of the biomass deconstruction may be compromised by diffusion limits of iron ions. By adopting genetic engineering to express the iron-binding protein (ferritin) or iron-binding peptide (IBP) in planta, we have successfully achieved iron accumulation in the biomass and enhanced pretreatability and saccharification of model plants by intracellular ferritin as FerIN [80], cell wall-bound IBP (IBPex) [81], and extracellular ferritin as FerEX [82]. It is noteworthy that our iron incorporation strategy in rice can also lead to a 35% increase in seed iron concentration and a 40% increase in seed yield, which lends promise to biotechnology of iron biofortification for sustainable agriculture [81]. These new approaches improved the economic and environmentally friendly aspects of the strategy by eliminating the extra step of soaking/spraying iron solution into the milled biomass and any treatment of extra iron solution.

Switchgrass has been identified as a target-sustainable bioenergy crop because it is a native grass species in the USA, can easily be integrated into conventional farming practices, and can be used as a forage crop. Moreover, switchgrass is a non-food crop that can grow in marginal land, which will not compete for agricultural land and food/grain market towards the biofuel or bioproduct production [17]. The high recalcitrance of this grass biomass, however, requires innovative technologies to facilitate its conversion [74, 105]. In this study, our main goal is to develop a more economical and environmentally friendly approach to reduce the recalcitrance of switchgrass biomass.

Building on our previous findings in the model plant systems, we developed a new genetic engineering strategy for iron incorporation by stacking both FerIN and IBPex into switchgrass. Our strategy increased the biomass yield (Fig. 4), iron content (Fig. 5), and iron binding ability of the switchgrass (Fig. 6). At the same time, the engineered switchgrass plants, especially FerIN/IBPex, have reduced recalcitrance and improved fermentable sugar yield after the HWP without additional harsh chemicals or exogenous iron supplement (Fig. 7). By coupling genetic iron incorporation approach with HWP, we demonstrated iron incorporation both intra- and extra-cellularly can be a promising approach to improve the biomass quality and conversion of switchgrass biomass [95, 101].

## Conclusion

In this study, we successfully transferred the metal catalyst platform to switchgrass using our consolidated switchgrass protocol. Several beneficial traits observed from our previous works in Arabidopsis and rice were represented in switchgrass biomass: FerIN transgenic switchgrass showed an increased number of tillers, biomass yield, and iron-binding ability. After stacking the cell wall targeting IBP to the cytosolic ferritin, FerIN/IBPex improved even further in height, number of tillers, biomass yield, and iron-binding ability. Using Prussian blue staining, the iron accumulation showed distinct distribution patterns that match our iron targeting strategy, cytosolic for FerIN, and both cytosolic and cell wall for FerEX transgenic lines. Finally, both FerIN and FerIN/IBPex transgenic switchgrass had improved in vitro iron-binding activity and in planta iron-accumulating ability, and their feedstock quality was also improved regarding pretreatability and digestibility.

We have provided a successful example for iron accumulation in switchgrass with improved biomass quality in terms of yield and saccharification by stacking intracellular ferritin and a cell wall-targeting IBP. It is noteworthy that the results demonstrated that iron incorporation can be applied as a universal approach to reduce barriers of thermochemical conversion and facilitate plant biomass deconstruction, even for highly recalcitrant species like switchgrass.

## Methods

### Chemicals and plant growth condition

All chemicals and plant materials for transgenic switchgrass production were following Lin et al. [43], and other chemicals, if not indicated specifically, were purchased from Sigma-Aldrich (St. Louis, MO). Plant materials were collected by following the standardized procedure described by Hardin et al. [106].

### DNA synthesis

The synthesized DNA fragments of IBP were designed as SP_GRP_-CBM11-IBP-6xHis (675 bp) and SP_GRP_-CBM11-4xIBP-6xHis (756 bp) flanking with *Pst*I and *Sac*I sites, which contains signal peptide of rice GRP (81 bp) from pCAMBIA1305.2 for apoplastic secretion in planta, CBM11 from *Clostridium thermocellum* (504 bp) for cellulose aiming, one or four tandem repeats of blood IBP sequence (108 bp) from Yang et al. [81] for iron-binding and followed by histidine tag (18 bp) for western blot detection.

### Construction, expression, and purification of iron-binding fusion proteins

Synthesized DNA fragments (SP_GRP_-CBM11-IBP-His_6_ and SP_GRP_-CBM11-4xIBP-His_6_) were cloned into a pET-22b (+) vector (Genscript, Piscataway, NJ) and transformed into *Escherichia coli* BL21 for protein expression. Starter cultures of each expression strain were inoculated into one liter of LB broth containing the appropriate antibiotic and grown at 37 °C until OD_600_ = 0.4. Cultures were induced with 0.25 mM IPTG and grown overnight at 17 °C.

The lysis of frozen cell pellets was conducted as described in Chung et al. except at room temperature [107]. The cell mixture was sonicated at room temperature for two min using a Branson 5510 water bath sonicator (Branson Ultrasonics Corporation, Danbury, CT). Centrifugation at 15,000×*g* for 20 min was performed to remove cell debris. The resulting supernatant in buffer A (50 mM Tris pH 8.0, 100 mM NaCl, 10 mM imidazole) was loaded onto a 5 mL HisTrap FF crude column (GE Healthcare, Piscataway, New Jersey, USA) on an AKTA FPLC (GE Healthcare, Piscataway, New Jersey, USA) and washed with buffer A. After washing, the protein was eluted with buffer B (50 mM Tris pH 8.0, 100 mM NaCl, and 200 mM imidazole). Affinity-purified proteins were further purified by size-exclusion chromatography using a HiLoad 16/600 Superdex 75 pg column (GE Healthcare, Piscataway, New Jersey, USA) in buffer C (50 mM Phosphate pH 7.0).

### Iron binding assay for purified iron-binding fusion proteins and crude protein extracts

For IBP-binding assay, we follow the methods described in Yang et al. [81]. The iron-binding abilities of single IBP and tandem repeat of IBP (4xIBP) were examined in two distinct pH environments, which is pH 5.5 and 7.0. The IBP-bound Fe in the supernatant was determined by orthophenanthroline, which results in a red solution when it binds with Fe^2+^ [108].

### Vector construction for transgenic switchgrass production

The overexpression of ferritin or blood iron-binding peptide (IBP) was achieved by cloning their coding sequence (CDS) into the corresponding pCAMBIA vectors. The intracellular ferritin-overexpressing (FerIN) vector, pCAMBIA-FerIN, was obtained from Hui et al. [80]. Two cloning steps were conducted for establishing extracellular IBP-overexpressing (IBPex) construct: (1) the *pporRFP* gene in pCAMBIA-RFP [43] was replaced by the synthesized IBPex fragment using *Pst*I and *Sac*I (Additional file 1: Figure S1), resulting in pCAMBIA-IBPex. (2) Then, the expression cassette of IBPex from pCAMBIA-IBPex was released by *Xba*I and *Pvu*II, and the stacking of FerIN and IBPex was achieved by inserting the IBPex cassette into pCAMBIA-FerIN, which is also digested by *Xba*I but partially by *Pvu*II. The two plasmids (pCAMBIA-FerIN and pCAMBIA-FerIN/IBPex) were introduced into *Agrobacterium* EHA105 by a freeze–thaw method [109].

### Transgenic switchgrass generation and genomic DNA PCR analysis of transgenic plants

The *Agrobacterium*-mediated genetic transformation and genomic DNA PCR were following our previously established method [43]. For genomic DNA PCR analysis, the primer sets for *hph* gene (D_Hph-F and D_Hph-R) are listed in Additional file 1: Table S1A.

### Southern blot analysis

The Southern blot analysis using fresh leaf tissue of EV, FerIN, and IBPex/FerIN followed our previously established method [43]. The genomic DNA of EV, FerIN, and IBPex/FerIN were digested with *Hind*III. The Southern blot analyses of the overnight-digested genomic DNA were performed according to Lin et al. (2017) using digoxigenin (DIG)-labeled *hph* fragment (745 bp) as the probe [43].

### Total RNA extraction, reverse transcription PCR (RT-PCR) and real-time quantitative RT-PCR (qRT-PCR)

The procedures of RNA extraction, reverse transcription PCR (RT-PCR) and real-time quantitative RT-PCR (qRT-PCR) followed our previously published methods from the E4 stage of WT or transgenic switchgrass plants [43]. The primer sets for detection of *hph*, *IBP* and ferritin are designed and listed in Additional file 1: Table S1B**,** and the RT-PCR reaction is following the genomic DNA PCR analysis. For qRT-PCR, the RNA was extracted from the transgene, and we extracted the RNA from greenhouse-grown switchgrass. The primer sets for *actin* and *ferritin* were derived from the literature [65, 80], while the primer set for *IBP* was designed in this study, which are all listed in Additional file 1: Table S1C. The qRT-PCR was conducted as previously described [110].

### Western blot analysis

The extraction of switchgrass total soluble protein was modified from Somleva et al. [111]. The stem tissue from the E4 stage of WT or transgenic switchgrass plants was snap-frozen in liquid nitrogen immediately after harvest and ground under liquid nitrogen into a fine powder before protein extraction. Three grams of stem powder was suspended in 5 mL of extraction buffer (100 mM sodium phosphate (pH 7.0), 10 mM EDTA, 20 mM sodium ascorbate, 4 mM β-mercaptoethanol, 0.1 mM phenylmethylsulphonyl fluoride (PMSF), 10% (w/w) polyvinylpolypyrrolidone and cOmplete™ EDTA-free Protease Inhibitor), homogenized on ice using an Ultra-Turrax T-18 basic disperser (IKA, Wilmington, NC), and spun at 4000×*g* at 4 °C for 15 min to remove cellular debris. The protein concentration of the extract was determined by the Bradford assay [112].

Twenty µg of total protein extracted from switchgrass transgenics were mixed with 4 × NuPAGE™ LDS sample buffer (NP0007, Thermo Fisher Scientific, Waltham, MA) and separated on Invitrogen NuPAGE Novex 4–12% Bis–Tris Mini Gels (NP0321BOX, Thermo Fisher Scientific**,** Waltham, MA), followed by transfer to a polyvinylidene difluoride (PVDF) membrane using the Invitrogen iBlot 2 gel transfer system (Thermo Fisher Scientific, Waltham, MA) and blocked using SuperBlock T20 PBS (Thermo Fisher Scientific Inc., Rockford, IL, USA) for 20 min. The western blot for detection of ferritin was performed using chicken IgY polyclonal antibody from Hui et al. [80] as the primary antibody and goat anti-chicken IgY (H+L) secondary antibody (Thermo Fisher Scientific, Waltham, MA), whereas the detection of IBP was performed using 6x-his tag monoclonal antibody (4A12E4) with alkaline phosphatase-conjugated rabbit anti-mouse IgG (H+L) (Thermo Fisher Scientific, Waltham, MA) as secondary. The alkaline phosphatase localization was visualized using 5-bromo-4-chloro-3′-indolylphosphate p-toluidine (BCIP)/ nitro-blue tetrazolium chloride (NBT) (Life Technologies Corp., Carlsbad, CA, USA).

### Determination of iron accumulation in shoot biomass of switchgrass plants

The switchgrass shoots at the R1 stage were harvested and rinsed three times with ddH_2_O so that no surface iron residues would affect the iron content measurement of biomass. Dry shoot samples were then ground to pass through a 20-mesh (1 mm) screen, and an aliquot of biomass powder was used to measure the iron concentration using the procedure modified from previous literature reports [82, 113–115]. Briefly, twenty micrograms of dry biomass powder were digested overnight at 70 °C with 0.4 mL 25% (v/v) nitric acid (Trace Metal Grade, Fisher Scientific). The acid extracts were diluted to 5 mL with fresh Millipore (Synergy water Purification System) de-ionized H_2_O (the final nitric acid concentration was 2%) and used for the iron concentration measurement using inductively coupled plasma/optical emission spectroscopy (ICP-OES) by the Chemical Analysis Laboratory at the University of Georgia.

### Perls’ Prussian blue iron staining

The Perls’ Prussian blue staining was performed using the R1 stage switchgrass stem cross-sections followed the procedure described in Hui et al. [80] and Yang et al. [81].

### Hot-water pretreatment (HWP) and co-saccharification of transgenic plant biomass

The stems from the above-ground R1 stage transgenic switchgrass were harvested by removing the inflorescence, leaf blades, sheaths, internode 1 (I1) and top of the tiller following the standardized protocol [106]. After air-drying in the greenhouse for 3 weeks, the stem of transgenic switchgrass was ground to pass through a 20-mesh (0.841 mm) screen using a Wiley Mini Mill (Thomas Scientific, Swedesboro, NJ). The milled material was then tested for total sugar release through a high-throughput method that combines hot-water pretreatment with enzymatic hydrolysis [116]. Briefly, 5 mg ground biomass was weighed in sample replicates into random individual wells on 96-well Hastelloy plates; ultrapure water (18.3 MΩ cm) from a MilliQ filter system was added. The plates were sealed with Teflon tape, clamped, and subjected to hot-water pretreatment at 180 °C for 17.5 min. The subsequent enzymatic saccharification was carried out by adding buffer to each well in the plate, mixing, and using Novozymes CTec2 at loadings of 70 mg enzyme/g biomass with incubation at 40 °C for 70 h. The sugar release was measured using a glucose oxidase–peroxidase (GOPOD) assay for glucose and a xylose dehydrogenase (XDH) assay for xylose absorbances versus standard curves [82].

### Statistical analysis

All experiments were conducted at least twice and all graphs and statistical analyzes were generated using Excel (Microsoft Inc., Redmond, WA) and SigmaPlot (SPSS Inc., Chicago, IL). Data are presented as mean (± SE) and the numbers of biological replicate for each experiment are indicated in the corresponding figure legends. Data were subjected to one-way analysis of variance (ANOVA) with Tukey’s post hoc test to analyze the significant differences between lines.

## Supplementary Information

**Additional file 1: Table S1.** Primer sets used for molecular analysis. **Figure S1.** Coding sequence (CDS) of SP_GRP_-CBM11-4xIBP (IBPex) fragment. **Figure S2**. The development of red coloration in the in vitro iron-binding assay. **Figure S3.** Colony PCR results from the *Agrobacteria* transformants. **Figure S4**. Detection of transgene gene expression in transgenic switchgrass lines using RT-PCR.

## Data Availability

The data supporting the conclusions of this article are included within the article.

## Abbreviations

SP: Signal peptide
GRP: Glycine-rich protein
CBM: Carbohydrate-binding module
IBP: Iron-binding peptide
Fer: Ferritin
FerIN: Intracellular ferritin
IBPex: Extracellular IBP
EV: Empty vector
SB: Southern blot
HWP: Hot-water pretreatment

## References

[1] Popp J, Lakner Z, Harangi-Rákos M, Fári M (2014). The effect of bioenergy expansion: Food, energy, and environment. Renew Sustain Energy Rev.

[2] Warner KJ, Jones GA (2017). A population-induced renewable energy timeline in nine world regions. Energy Policy.

[3] IRENA (2018). Global energy transformation: a roadmap to 2050.

[4] Marinas MC, Dinu M, Socol AG, Socol C (2018). Renewable energy consumption and economic growth. Causality relationship in central and eastern European countries. PLoS ONE.

[5] Xie F, Liu C, Chen H, Wang N (2018). Threshold effects of new energy consumption transformation on economic growth. Sustainability.

[6] Yuan JS, Tiller KH, Al-Ahmad H, Stewart NR, Stewart CN (2008). Plants to power: bioenergy to fuel the future. Trends Plant Sci.

[7] Caspeta L, Buijs NAA, Nielsen J (2013). The role of biofuels in the future energy supply. Energy Environ Sci.

[8] Ben-Iwo J, Manovic V, Longhurst P (2016). Biomass resources and biofuels potential for the production of transportation fuels in Nigeria. Renew Sustain Energy Rev.

[9] Halford NG, Karp A (2010). Energy crops.

[10] Gavrilescu M, Gupta VK, Tuohy MG, Kubicek CP, Saddler J, Xu F (2014). Biorefinery systems: an overview. Bioenergy research: advances and applications.

[11] Loqué D, Scheller HV, Pauly M (2015). Engineering of plant cell walls for enhanced biofuel production. Curr Opin Plant Biol.

[12] Amore A, Ciesielski PN, Lin C-Y, Salvachúa D (2016). Sànchez i Nogué V: Development of lignocellulosic biorefinery technologies: recent advances and current challenges. Aust J Chem.

[13] Carroll A, Somerville C (2009). Cellulosic biofuels. Annu Rev Plant Biol.

[14] Naik SN, Goud VV, Rout PK, Dalai AK (2010). Production of first and second generation biofuels: a comprehensive review. Renew Sustain Energy Rev.

[15] Hirani AH, Javed N, Asif M, Basu SK, Kumar A, Kumar A, Ogita S, Yau Y-Y (2018). A review on first- and second-generation biofuel productions. Biofuels: greenhouse gas mitigation and global warming: next generation biofuels and role of biotechnology.

[16] Nageswara-Rao M, Soneji JR, Kwit C, Stewart CN (2013). Advances in biotechnology and genomics of switchgrass. Biotechnol Biofuels.

[17] McLaughlin SB, Adams Kszos L (2005). Development of switchgrass (*Panicum virgatum*) as a bioenergy feedstock in the United States. Biomass Bioenerg.

[18] Brosse N, Dufour A, Meng X, Sun Q, Ragauskas A (2012). *Miscanthus*: a fast-growing crop for biofuels and chemicals production. Biofuels Bioprod Biorefin.

[19] Mathur S, Umakanth AV, Tonapi VA, Sharma R, Sharma MK (2017). Sweet sorghum as biofuel feedstock: recent advances and available resources. Biotechnol Biofuels.

[20] Sannigrahi P, Ragauskas AJ, Tuskan GA (2010). Poplar as a feedstock for biofuels: a review of compositional characteristics. Biofuels Bioprod Biorefin.

[21] Krzyżaniak M, Stolarski MJ, Waliszewska B, Szczukowski S, Tworkowski J, Załuski D, Śnieg M (2014). Willow biomass as feedstock for an integrated multi-product biorefinery. Ind Crops Prod.

[22] Albersheim P, Darvill A, Roberts K, Sederoff R, Staehelin A (2010). Plant cell walls.

[23] Zeng Y, Himmel ME, Ding S-Y (2017). Visualizing chemical functionality in plant cell walls. Biotechnol Biofuels.

[24] Kumar P, Barrett DM, Delwiche MJ, Stroeve P (2009). Methods for pretreatment of lignocellulosic biomass for efficient hydrolysis and biofuel production. Ind Eng Chem Res.

[25] Sluiter A, Hames B, Ruiz R, Scarlata C, Sluiter J, Templeton D, Crocker D: Determination of structural carbohydrates and lignin in biomass. Laboratory analytical procedure 2012: NREL/TP-510–42618.

[26] Kim JS, Lee YY, Kim TH (2016). A review on alkaline pretreatment technology for bioconversion of lignocellulosic biomass. Biores Technol.

[27] Teymouri F, Laureano-Perez L, Alizadeh H, Dale BE (2005). Optimization of the ammonia fiber explosion (AFEX) treatment parameters for enzymatic hydrolysis of corn stover. Biores Technol.

[28] Jacquet N, Maniet G, Vanderghem C, Delvigne F, Richel A (2015). Application of steam explosion as pretreatment on lignocellulosic material: a review. Ind Eng Chem Res.

[29] Zhuang X, Wang W, Yu Q, Qi W, Wang Q, Tan X, Zhou G, Yuan Z (2016). Liquid hot water pretreatment of lignocellulosic biomass for bioethanol production accompanying with high valuable products. Biores Technol.

[30] Kumar P, Barrett DM, Delwiche MJ, Stroeve P (2011). Pulsed electric field pretreatment of switchgrass and wood chip species for biofuel production. Ind Eng Chem Res.

[31] Golberg A, Sack M, Teissie J, Pataro G, Pliquett U, Saulis G, Stefan T, Miklavcic D, Vorobiev E, Frey W (2016). Energy-efficient biomass processing with pulsed electric fields for bioeconomy and sustainable development. Biotechnol Biofuels.

[32] Ciesielski PN, Resch MG, Hewetson B, Killgore JP, Curtin A, Anderson N, Chiaramonti AN, Hurley DC, Sanders A, Himmel ME (2014). Engineering plant cell walls: tuning lignin monomer composition for deconstructable biofuel feedstocks or resilient biomaterials. Green Chem.

[33] Boufi S (2017). Agricultural crop residue as a source for the production of cellulose nanofibrils. Cellulose-reinforced nanofibre composites.

[34] Himmel ME, Ding S-Y, Johnson DK, Adney WS, Nimlos MR, Brady JW, Foust TD (2007). Biomass recalcitrance: engineering plants and enzymes for biofuels production. Science.

[35] Zeng Y, Zhao S, Yang S, Ding S-Y (2014). Lignin plays a negative role in the biochemical process for producing lignocellulosic biofuels. Curr Opin Biotechnol.

[36] Mahon EL, Mansfield SD (2019). Tailor-made trees: engineering lignin for ease of processing and tomorrow’s bioeconomy. Curr Opin Biotechnol.

[37] McLaughlin S, Bouton J, Bransby D, Conger B, Ocumpaugh W, Parrish D, Taliaferro C, Vogel K, Wullschleger S (1999). Developing switchgrass as a bioenergy crop. Perspect N Crops N Uses.

[38] Cox C, Garrett K, Bockus W (2005). Meeting the challenge of disease management in perennial grain cropping systems. Renewable Agric Food Syst.

[39] Wright L. Historical perspective on how and why switchgrass was selected as a “model” high-potential energy crop; 2007.

[40] Casler MD, Tobias CM, Kaeppler SM, Buell CR, Wang Z-Y, Cao P, Schmutz J, Ronald P (2011). The switchgrass genome: tools and strategies. Plant Genome.

[41] Fike JH, Pease JW, Owens VN, Farris RL, Hansen JL, Heaton EA, Hong CO, Mayton HS, Mitchell RB, Viands DR (2017). Switchgrass nitrogen response and estimated production costs on diverse sites. GCB Bioenergy.

[42] Hendrickson J, Schmer MR, Sanderson MA (2013). Water use efficiency by switchgrass compared to a native grass or a native grass alfalfa mixture. BioEnergy Res.

[43] Lin CY, Donohoe BS, Ahuja N, Garrity DM, Qu R, Tucker MP, Himmel ME, Wei H (2017). Evaluation of parameters affecting switchgrass tissue culture: toward a consolidated procedure for *Agrobacterium*-mediated transformation of switchgrass (*Panicum virgatum*). Plant Methods.

[44] Pedroso GM, De Ben C, Hutmacher RB, Orloff S, Putnam D, Six J, van Kessel C, Wright SD, Linquist B (2011). Switchgrass is a promising, high-yielding crop for California biofuel. Calif Agric.

[45] Guretzky JA, Biermacher JT, Cook BJ, Kering MK, Mosali J (2011). Switchgrass for forage and bioenergy: harvest and nitrogen rate effects on biomass yields and nutrient composition. Plant Soil.

[46] O'Bryan PJ, Hector RE, Iten LB, Mitchell RB, Qureshi N, Sarath G, Vogel KP, Cotta MA (2013). Conversion of switchgrass to ethanol using dilute ammonium hydroxide pretreatment: influence of ecotype and harvest maturity AU – Dien, Bruce S. Environ Technol.

[47] Ashworth AJ, Sadaka SS, Allen FL, Sharara MA, Keyser PD (2014). Influence of pyrolysis temperature and production conditions on switchgrass biochar for use as a soil amendment. BioResources.

[48] Karp EM, Resch MG, Donohoe BS, Ciesielski PN, O’Brien MH, Nill JE, Mittal A, Biddy MJ, Beckham GT (2015). Alkaline pretreatment of switchgrass. ACS Sustain Chem Eng.

[49] Bahri BA, Daverdin G, Xu X, Cheng J-F, Barry KW, Brummer EC, Devos KM (2018). Natural variation in genes potentially involved in plant architecture and adaptation in switchgrass (*Panicum virgatum* L.). BMC Evol Biol.

[50] Abramson M, Shoseyov O, Shani Z (2010). Plant cell wall reconstruction toward improved lignocellulosic production and processability. Plant Sci.

[51] Xi Y, Fu C, Ge Y, Nandakumar R, Hisano H, Bouton J, Wang Z-Y (2009). *Agrobacterium*-mediated transformation of switchgrass and inheritance of the transgenes. Bioenergy Res.

[52] Chen F, Dixon RA (2007). Lignin modification improves fermentable sugar yields for biofuel production. Nat Biotechnol.

[53] Li X, Weng J-K, Chapple C (2008). Improvement of biomass through lignin modification. Plant J.

[54] Wang JP, Matthews ML, Williams CM, Shi R, Yang C, Tunlaya-Anukit S, Chen H-C, Li Q, Liu J, Lin C-Y (2018). Improving wood properties for wood utilization through multi-omics integration in lignin biosynthesis. Nat Commun.

[55] Zhao Q, Nakashima J, Chen F, Yin Y, Fu C, Yun J, Shao H, Wang X, Wang Z-Y, Dixon RA (2013). Laccase is necessary and nonredundant with peroxidase for lignin polymerization during vascular development in *Arabidopsis*. Plant Cell.

[56] Lin C-Y, Li Q, Tunlaya-Anukit S, Shi R, Sun Y-H, Wang JP, Liu J, Loziuk P, Edmunds CW, Miller ZD (2016). A cell wall-bound anionic peroxidase, PtrPO21, is involved in lignin polymerization in *Populus trichocarpa*. Tree Genet Genomes.

[57] Eudes A, George A, Mukerjee P, Kim JS, Pollet B, Benke PI, Yang F, Mitra P, Sun L, Çetinkol ÖP (2012). Biosynthesis and incorporation of side-chain-truncated lignin monomers to reduce lignin polymerization and enhance saccharification. Plant Biotechnol J.

[58] Eudes A, Sathitsuksanoh N, Baidoo EEK, George A, Liang Y, Yang F, Singh S, Keasling JD, Simmons BA, Loqué D (2015). Expression of a bacterial 3-dehydroshikimate dehydratase reduces lignin content and improves biomass saccharification efficiency. Plant Biotechnol J.

[59] Eudes A, Berthomieu R, Hao Z, Zhao N, Benites VT, Baidoo EE, Loqué D (2018). Production of muconic acid in plants. Metab Eng.

[60] Li C, Wang JPY, Nishimura Y, Li Q, Chiang VL, Horvath B, Min D, Jameel H, Chang HM, Peszlen I, Horvath L (2011). Down-regulation of glycosyltransferase 8D genes in *Populus trichocarpa* caused reduced mechanical strength and xylan content in wood. Tree Physiol.

[61] Chiniquy D, Sharma V, Schultink A, Baidoo EE, Rautengarten C, Cheng K, Carroll A, Ulvskov P, Harholt J, Keasling JD (2012). XAX1 from glycosyltransferase family 61 mediates xylosyltransfer to rice xylan. Proc Natl Acad Sci.

[62] Vega-Sánchez ME, Loqué D, Lao J, Catena M, Verhertbruggen Y, Herter T, Yang F, Harholt J, Ebert B, Baidoo EEK (2015). Engineering temporal accumulation of a low recalcitrance polysaccharide leads to increased C6 sugar content in plant cell walls. Plant Biotechnol J.

[63] Biswal AK, Atmodjo MA, Li M, Baxter HL, Yoo CG, Pu Y, Lee Y-C, Mazarei M, Black IM, Zhang J-Y (2018). Sugar release and growth of biofuel crops are improved by downregulation of pectin biosynthesis. Nat Biotechnol.

[64] Saathoff AJ, Sarath G, Chow EK, Dien BS, Tobias CM (2011). Downregulation of cinnamyl-alcohol dehydrogenase in switchgrass by RNA silencing results in enhanced glucose release after cellulase treatment. PLoS ONE.

[65] Xu B, Escamilla-Treviño LL, Sathitsuksanoh N, Shen Z, Shen H, Percival Zhang Y-H, Dixon RA, Zhao B (2011). Silencing of 4-coumarate:coenzyme A ligase in switchgrass leads to reduced lignin content and improved fermentable sugar yields for biofuel production. New Phytol.

[66] Fu C, Mielenz JR, Xiao X, Ge Y, Hamilton CY, Rodriguez M, Chen F, Foston M, Ragauskas A, Bouton J (2011). Genetic manipulation of lignin reduces recalcitrance and improves ethanol production from switchgrass. Proc Natl Acad Sci.

[67] Shen H, He X, Poovaiah CR, Wuddineh WA, Ma J, Mann DGJ, Wang H, Jackson L, Tang Y, Neal Stewart Jr C (2012). Functional characterization of the switchgrass (*Panicum virgatum*) R2R3-MYB transcription factor PvMYB4 for improvement of lignocellulosic feedstocks. New Phytol.

[68] Shen H, Poovaiah CR, Ziebell A, Tschaplinski TJ, Pattathil S, Gjersing E, Engle NL, Katahira R, Pu Y, Sykes R (2013). Enhanced characteristics of genetically modified switchgrass (*Panicum virgatum* L.) for high biofuel production. Biotechnol Biofuels.

[69] Gallego-Giraldo L, Shadle G, Shen H, Barros-Rios J, Fresquet Corrales S, Wang H, Dixon RA (2016). Combining enhanced biomass density with reduced lignin level for improved forage quality. Plant Biotechnol J.

[70] Wuddineh WA, Mazarei M, Turner GB, Sykes RW, Decker SR, Davis MF, Stewart CN (2015). Identification and molecular characterization of the switchgrass AP2/ERF transcription factor superfamily, and overexpression of PvERF001 for improvement of biomass characteristics for biofuel. Front Bioeng Biotechnol.

[71] Wuddineh WA, Mazarei M, Zhang J-Y, Turner GB, Sykes RW, Decker SR, Davis MF, Udvardi MK, Stewart CN (2016). Identification and overexpression of a Knotted1-like transcription factor in switchgrass (*Panicum virgatum* L.) for lignocellulosic feedstock improvement. Front Plant Sci.

[72] Wu Z, Cao Y, Yang R, Qi T, Hang Y, Lin H, Zhou G, Wang Z-Y, Fu C (2016). Switchgrass SBP-box transcription factors PvSPL1 and 2 function redundantly to initiate side tillers and affect biomass yield of energy crop. Biotechnol Biofuels.

[73] Yan J, Liu Y, Wang K, Li D, Hu Q, Zhang W (2018). Overexpression of *OsPIL1* enhanced biomass yield and saccharification efficiency in switchgrass. Plant Sci.

[74] Li G, Jones KC, Eudes A, Pidatala VR, Sun J, Xu F, Zhang C, Wei T, Jain R, Birdseye D (2018). Overexpression of a rice BAHD acyltransferase gene in switchgrass (*Panicum virgatum* L.) enhances saccharification. BMC Biotechnol.

[75] Chuck GS, Tobias C, Sun L, Kraemer F, Li C, Dibble D, Arora R, Bragg JN, Vogel JP, Singh S (2011). Overexpression of the maize Corngrass1 microRNA prevents flowering, improves digestibility, and increases starch content of switchgrass. Proc Natl Acad Sci.

[76] Fu C, Sunkar R, Zhou C, Shen H, Zhang J-Y, Matts J, Wolf J, Mann DGJ, Stewart CN, Tang Y, Wang Z-Y (2012). Overexpression of miR156 in switchgrass (*Panicum virgatum* L.) results in various morphological alterations and leads to improved biomass production. Plant Biotechnol J.

[77] Gilna P, Lynd LR, Mohnen D, Davis MF, Davison BH (2017). Progress in understanding and overcoming biomass recalcitrance: a BioEnergy Science Center (BESC) perspective. Biotechnol Biofuels.

[78] Nguyen QA, Tucker MP: Dilute acid/metal salt hydrolysis of lignocellulosics. Google Patents; 2002.

[79] Wei H, Donohoe BS, Vinzant TB, Ciesielski PN, Wang W, Gedvilas LM, Zeng Y, Johnson DK, Ding SY, Himmel ME, Tucker MP (2011). Elucidating the role of ferrous ion cocatalyst in enhancing dilute acid pretreatment of lignocellulosic biomass. Biotechnol Biofuels.

[80] Wei H, Yang H, Ciesielski PN, Donohoe BS, McCann MC, Murphy AS, Peer WA, Ding S-Y, Himmel ME, Tucker MP (2015). Transgenic ferritin overproduction enhances thermochemical pretreatments in *Arabidopsis*. Biomass Bioenerg.

[81] Yang H, Wei H, Ma G, Antunes MS, Vogt S, Cox J, Zhang X, Liu X, Bu L, Gleber SC (2016). Cell wall targeted *in planta* iron accumulation enhances biomass conversion and seed iron concentration in *Arabidopsis* and rice. Plant Biotechnol J.

[82] Lin CY, Jakes JE, Donohoe BS, Ciesielski PN, Yang H, Gleber SC, Vogt S, Ding SY, Peer WA, Murphy AS (2016). Directed plant cell-wall accumulation of iron: embedding co-catalyst for efficient biomass conversion. Biotechnol Biofuels.

[83] Beasley JT, Bonneau JP, Sánchez-Palacios JT, Moreno-Moyano LT, Callahan DL, Tako E, Glahn RP, Lombi E, Johnson AAT (2019). Metabolic engineering of bread wheat improves grain iron concentration and bioavailability. Plant Biotechnol J.

[84] Fang R-X, Pang Z, Gao D-M, Mang K-Q, Chua N-H (1991). cDNA sequence of a virus-inducible, glycine-rich protein gene from rice. Plant Mol Biol.

[85] Chen X, Equi R, Baxter H, Berk K, Han J, Agarwal S, Zale J (2010). A high-throughput transient gene expression system for switchgrass (*Panicum virgatum* L.) seedlings. Biotechnol Biofuels.

[86] Kumar V, Campbell LM, Rathore KS (2011). Rapid recovery- and characterization of transformants following *Agrobacterium*-mediated T-DNA transfer to sorghum. Plant Cell Tissue Organ Cult (PCTOC).

[87] Wei H, Chen X, Shekiro J, Kuhn E, Wang W, Ji Y, Kozliak E, Himmel M, Tucker M (2018). Kinetic modelling and experimental studies for the effects of Fe^2+^ Ions on xylan hydrolysis with dilute-acid pretreatment and subsequent enzymatic hydrolysis. Catalysts.

[88] De Loose M, Gheysen G, Tire C, Gielen J, Villarroel R, Genetello C, Van Montagu M, Depicker A, Inzé D (1991). The extensin signal peptide allows secretion of a heterologous protein from protoplasts. Gene.

[89] Viegas A, Sardinha J, Freire F, Duarte Daniel F, Carvalho Ana L, Fontes Carlos MGA, Romão Maria J, Macedo Anjos L, Cabrita Eurico J (2013). Solution structure, dynamics and binding studies of a family 11 carbohydrate-binding module from *Clostridium thermocellum* (CtCBM11). Biochem J.

[90] Lee S-H, Song KB (2009). Purification of an iron-binding nona-peptide from hydrolysates of porcine blood plasma protein. Process Biochem.

[91] Gelvin SB (2003). *Agrobacterium*-mediated plant transformation: the biology behind the "gene-jockeying" tool. Microbiol Mol Biol Rev MMBR.

[92] Bubner B, Gase K, Baldwin IT (2004). Two-fold differences are the detection limit for determining transgene copy numbers in plants by real-time PCR. BMC Biotechnol.

[93] Ting-Bo J, Bao-Jian D, Feng-Juan LI, Chuan-Ping Y (2006). Differential expression of endogenous ferritin genes and iron homeostasis alteration in transgenic tobacco overexpressing soybean ferritin gene. Acta Genetica Sinica.

[94] Arosio P, Ingrassia R, Cavadini P (2009). Ferritins: a family of molecules for iron storage, antioxidation and more. Biochimica et Biophysica Acta BBA General Subjects.

[95] Briat J-F, Dubos C, Gaymard F (2015). Iron nutrition, biomass production, and plant product quality. Trends Plant Sci.

[96] Guo A, Hu Y, Shi M, Wang H, Wu Y, Wang Y (2020). Effects of iron deficiency and exogenous sucrose on the intermediates of chlorophyll biosynthesis in *Malus halliana*. PLoS ONE.

[97] Goto F, Yoshihara T, Saiki H (2000). Iron accumulation and enhanced growth in transgenic lettuce plants expressing the iron- binding protein ferritin. Theor Appl Genet.

[98] Hegedűs A, Janda T, Horváth GV, Dudits D (2008). Accumulation of overproduced ferritin in the chloroplast provides protection against photoinhibition induced by low temperature in tobacco plants. J Plant Physiol.

[99] Parveen S, Gupta DB, Dass S, Kumar A, Pandey A, Chakraborty S, Chakraborty N (2016). Chickpea ferritin CaFer1 participates in oxidative stress response and promotes growth and development. Sci Rep.

[100] Frederick N, Li M, Carrier DJ, Buser MD, Wilkins MR (2016). Switchgrass storage effects on the recovery of carbohydrates after liquid hot water pretreatment and enzymatic hydrolysis. AIMS Bioeng.

[101] Yan L, Ma R, Li L, Fu J (2016). Hot water pretreatment of lignocellulosic biomass: An effective and environmentally friendly approach to enhance biofuel production. Chem Eng Technol.

[102] Martín-Lara MA, Chica-Redecillas L, Pérez A, Blázquez G, Garcia-Garcia G, Calero M (2020). Liquid hot water pretreatment and enzymatic hydrolysis as a valorization route of Italian green pepper waste to delivery free sugars. Foods.

[103] Shen B, Sun X, Zuo X, Shilling T, Apgar J, Ross M, Bougri O, Samoylov V, Parker M, Hancock E (2012). Engineering a thermoregulated intein-modified xylanase into maize for consolidated lignocellulosic biomass processing. Nat Biotechnol.

[104] Mitros T, Session AM, James BT, Wu GA, Belaffif MB, Clark LV, Shu S, Dong H, Barling A, Holmes JR (2020). Genome biology of the paleotetraploid perennial biomass crop *Miscanthus*. Nat Commun.

[105] Vogel J (2008). Unique aspects of the grass cell wall. Curr Opin Plant Biol.

[106] Hardin CF, Fu C, Hisano H, Xiao X, Shen H, Stewart CN, Parrott W, Dixon RA, Wang Z-Y (2013). Standardization of switchgrass sample collection for cell wall and biomass trait analysis. BioEnergy Res.

[107] Chung D, Sarai NS, Knott BC, Hengge N, Russell JF, Yarbrough JM, Brunecky R, Young J, Supekar N, Vander Wall T (2019). Glycosylation is vital for industrial performance of hyperactive cellulases. ACS Sustain Chem Eng.

[108] Saywell LG, Cunningham BB (1937). Determination of Iron: colorimetric *o*-phenanthroline method. Ind Eng Chem Anal Ed.

[109] Weigel D, Glazebrook J (2006). Transformation of *Agrobacterium* using the freeze-thaw method. CSH Protoc.

[110] Zhao S, Wei H, Lin C-Y, Zeng Y, Tucker MP, Himmel ME, Ding S-Y (2016). *Burkholderia phytofirmans* inoculation-induced changes on the shoot cell anatomy and iron accumulation reveal novel components of arabidopsis-endophyte interaction that can benefit downstream biomass deconstruction. Front Plant Sci.

[111] Somleva MN, Snell KD, Beaulieu JJ, Peoples OP, Garrison BR, Patterson NA (2008). Production of polyhydroxybutyrate in switchgrass, a value-added co-product in an important lignocellulosic biomass crop. Plant Biotechnol J.

[112] Bradford MM (1976). A rapid and sensitive method for the quantitation of microgram quantities of protein utilizing the principle of protein-dye binding. Anal Biochem.

[113] Wei H, Layzell DB (2006). Adenylate-coupled ion movement. A mechanism for the control of nodule permeability to O2 diffusion. Plant Physiol.

[114] Vansuyt G, Robin A, Briat J-F, Curie C, Lemanceau P (2007). Iron acquisition from Fe-pyoverdine by *Arabidopsis thaliana*. Mol Plant Microbe Interact.

[115] Stacey MG, Patel A, McClain WE, Mathieu M, Remley M, Rogers EE, Gassmann W, Blevins DG, Stacey G (2008). The *Arabidopsis* AtOPT3 protein functions in metal homeostasis and movement of iron to developing seeds. Plant Physiol.

[116] Selig MJ, Tucker MP, Sykes RW, Reichel KL, Brunecky R, Himmel ME, Davis MF, Decker SR (2010). Lignocellulose recalcitrance screening by integrated high-throughput hydrothermal pretreatment and enzymatic saccharification. Ind Biotechnol.

