# Supplementary material for: Iron incorporation both intra- and extra-cellularly improves the yield and saccharification of switchgrass (Panicum virgatum L.) biomass
Source: Biotechnol Biofuels. 2021 Mar 4;14:55. doi: 10.1186/s13068-021-01891-4 (PMC7931346; doi:10.1186/s13068-021-01891-4)
Supplement: Supplementary file 1 — Additional file 1: Table S1. Primer sets used for molecular analysis. Figure S1. Coding sequence (CDS) of SPGRP-CBM11-4xIBP (IBPex) fragment. Figure S2. The development of red coloration in the in vitro iron-binding assay. Figure S3. Colony PCR results from the Agrobacteria transformants. Figure S4. Detection of transgene gene expression in transgenic switchgrass lines using RT-PCR. [file 13068_2021_1891_MOESM1_ESM.docx]

**Manuscript title:**

**Iron incorporation both intra- and extra-cellularly improves the yield and saccharification of switchgrass (*Panicum virgatum* L.) biomass**

**Additional file: Additional table and figures.**

**Contents:**

- **Table S1.** Primer sets used for molecular analysis.
- **Figure S1.** Coding sequence (CDS) of SP_GRP_-CBM11-4xIBP (IBPex) fragment.
- **Figure S2**. The development of red coloration in the *in vitro* iron-binding assay.
- **Figure S3.** Colony PCR results from the *Agrobacteria* transformants.
- **Figure S4**. Detection of transgene gene expression in transgenic switchgrass lines using RT-PCR.

| **Table S1**. Primer sets used for molecular analysis.  **A. Genomic DNA and colony PCR** | | | | |
| --- | --- | --- | --- | --- |
| **Name** | **Sequence (5' to 3')** | **Target** | **Amplicon (bp)** | **Reference** |
| D_Hph-F | CGTTATGTTTATCGGCACTTTGCAT | Hph | 950 | Lin et al (2017) |
| D_Hph-R | AGCGAAACCCTATAGGAACCCTAAT |  |  |  |
| D_IBP-F | ACACTGGGTTTACTCAATAA | IBP | 879 | This study |
| D_IBP-R | AGGTTTCCCGACTGGAAAGC |  |  |  |
| D_Ferritin-F | ACCCACGAGGAGCATCGTGG | Ferritin | 811 | This study |
| D_Ferritin -R | AGCTGGTCACCTTATCAATCTAAC |  |  |  |
| **B. Reverse transcription PCR (RT-PCR)** | | | | |
| **Name** | **Sequence (5' to 3')** | **Target** | **Amplicon (bp)** | **Reference** |
| RT_Hph-F | CAACCAAGCTCTGATAGAGT | Hph | 658 | This study |
| RT_Hph-R | CGTTATGTTTATCGGCACTTTGCAT |  |  |  |
| RT_IBP-F | TACTCCGGTGAAGGTGCAAA | IBP | 358 | This study |
| RT_IBP-R | CCAAAGTACCGCTCATATCCTG |  |  |  |
| RT_Ferritin-F | TCTGAGTACCGTGCCTTTGAC | Ferritin | 321 | This study |
| RT_Ferritin-R | GAGCACAACCCTACCACCTC |  |  |  |

| **C. Real-time quantitative RT-PCR (qRT-PCR)** | | | | |
| --- | --- | --- | --- | --- |
| **Name** | **Sequence (5' to 3')** | **Target** | **Amplicon (bp)** | **Reference** |
| qRT_Ferritin-F | GAATACAACGCTTCATATGTGTACCA | Ferritin | 120 | Hui et al (2015) |
| qRT_Ferritin-R | AGCGTGCTCCCTTTCCTCTT |  |  |  |
| qRT_IBP-F | TACTCCGGTGAAGGTGCAAA | IBP | 70 | This study |
| qRT_IBP-R | TGACTTCCATGCCGTTTCCT |  |  |  |
| qRT_ACTIN2-F | GCGAGCTTCCCTGTAGGTA | Actin | 93 | Xu et al (2011) |
| qRT_ACTIN2-R | GTATGGTGAAGGCTGGGTTCG |  |  |  |


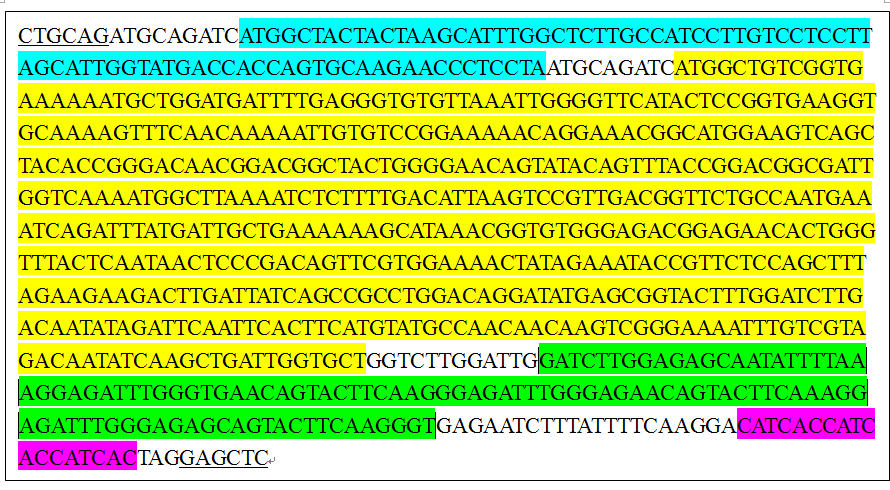


**Figure S1**. Coding sequence (CDS) of SP_GRP_-CBM11-4xIBP (IBPex) fragment. GRP signal sequence is highlighted in light blue, the CBM11 from *Clostridium thermocellum* is highlighted in yellow, the 4xIBP is highlighted in green, the 6xHis tag is highlighted in magenta, and the restriction enzymes (*Pst*I and *Sac*I) sites are underlined.

**
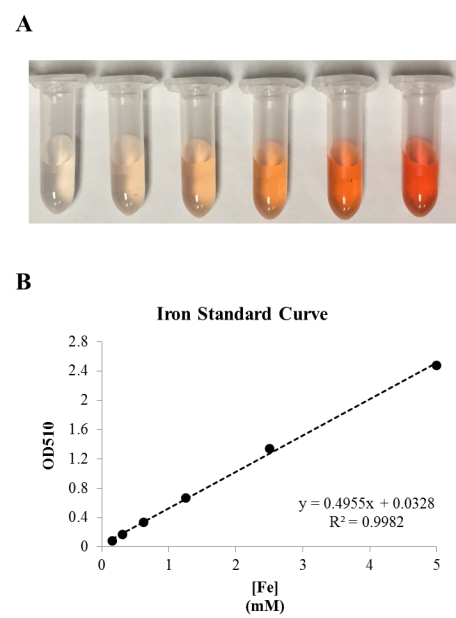
**

**Figure S2**. The development of red coloration in the *in vitro* iron-binding assay. (**A**) Serial dilution of the iron ion-containing solution in an arrangement of low to the high iron concentration (left to the right). (**B**) The standard curve for the iron-binding assay.


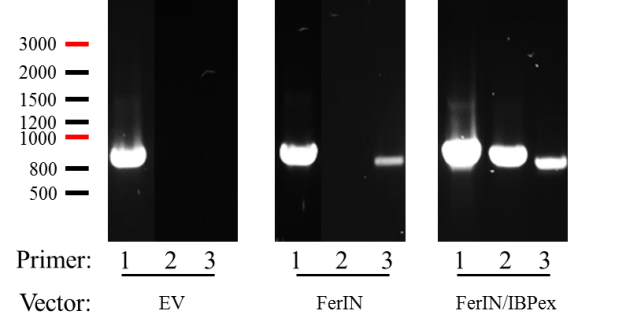


**Figure S3.** Colony PCR results from the *Agrobacteria* transformants. Primer set 1 (D_Hph-F and D_Hph-R) is specific for the *hph* gene. Primer set 2 (D_IBP -F and D_ IBP-R is for the *IBP* gene. Primer set 3 (D_Ferritin-F and D_Ferritin-R) is for the ferritin gene. **EV,** pCAMBIA-EV; **FerIN,** pCAMBIA-FerIN; **FerIN/IBPex,** pCAMBIA-FerIN/IBPex.


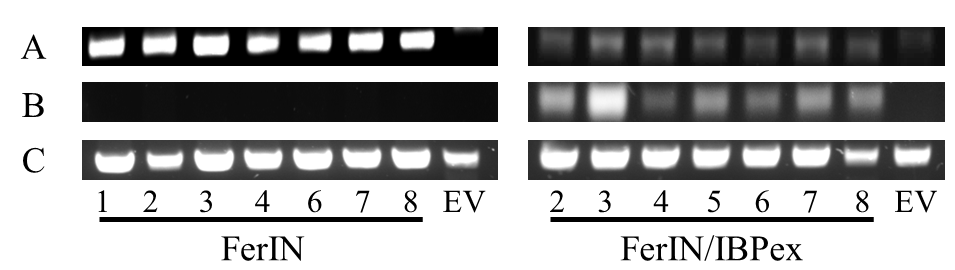


**Figure S4.** Detection of transgene gene expression in transgenic switchgrass lines using RT-PCR. (**A**) Primer set for ferritin (RT_ Ferritin –F and RT_ Ferritin -R). (**B**) Primer set for IBP (RT_IBP-F and RT_IBP-R). (**C**) Primer set for hygromycin (*hgh*) (RT_Hph-F and RT_Hph-R).

**References**

- Wei H, Yang H, Ciesielski PN, Donohoe BS, McCann MC, Murphy AS, Peer WA, Ding S-Y, Himmel ME, Tucker MP: Transgenic ferritin overproduction enhances thermochemical pretreatments in Arabidopsis. Biomass and Bioenergy 2015, 72:55-64.
- Xu B, Escamilla-Trevino LL, Sathitsuksanoh N, Shen Z, Shen H, Zhang YH, Dixon RA, Zhao B: Silencing of 4-coumarate:coenzyme A ligase in switchgrass leads to reduced lignin content and improved fermentable sugar yields for biofuel production. New Phytol 2011, 192:611-625.
- Lin CY, Donohoe BS, Ahuja N, Garrity DM, Qu R, Tucker MP, Himmel ME, Wei H: Evaluation of parameters affecting switchgrass tissue culture: toward a consolidated procedure for Agrobacterium-mediated transformation of switchgrass (*Panicum virgatum*). Plant Methods 2017, 13:113.
